# Guy's Hospital

**Published:** 1903-07-11

**Authors:** 


### GUY'S HOSPITAL.

Saturday, July 4th, was an eventful day at Guy's Hospital, the proceedings including the unveiling of the South African War memorial, the opening of the Wills Library, the distribution of prizes to the students and a large garden party. At 2.15 took place the unveiling of the war memorial by General Sir Richard Harrison. The memorial, which has been subscribed for by Guy's men and their friends, takes the form of a drinking fountain and has been placed in the colonnade of the hospital immediately to the south of the doorway into the Astley Cooper Ward. It is 6 feet 6 inches wide and 7 feet 6 inches high, the design being classic. The base is of Sienna marble, the columns on either side in Verte-Antico, with gun-metal Ionic capitals, and the entablature in Pavanazza. The pediment is surmounted by the arms of the hospital in bronze and enamel. Over the fountain is a canopy supported by cherubs and bearing the following inscription in gun-metal:—"To the Guy's men who died in the South African War, 1899–1901. Ante Diem Perierunt sed Militantes sed pro Patria." The names of those commemorated are arranged in the order of the dates at which they entered the hospital as students. They are as follows:—Thomas Jones, superintendent of the Welsh Army Hospital, died at Springfontein; Quinten Reid Veitch, Major Cape Medical Staff Corps, died at Capetown; Charles Pope Walker, Major Royal Army Medical Corps, died in Ladysmith during siege; Frederick Murray Russell, Captain New Zealand Contingent, killed in action at Rhenoster Kop; Hugh Arnold Bryant, Civil Surgeon, died at Bloemfontein; Francis Wellford, Surgeon Captain 7th Imperial Yeomanry, killed at the battle of Vlakfontein; Stanley Whicher, Civil Surgeon Natal Field Force, died at Mooi River; Richard Truman Fitz-Hugh, Civil Surgeon attached to the Imperial Yeomanry Hospital, Deelfontein, died there; Hugh Bernard-Onraet, Lieutenant Royal Army Medical Corps, killed at the battle of Pieter's Hill on the relief of Ladysmith; Charles Bernard Sells, Surgical Assistant at the Imperial Yeomanry Hospital, Deelfontein, where he died; Lawson Jervis Hughes, Mounted Medical Orderly Imperial Yeomanry Field Hospital, killed in action at Kroonstad, on refusing to surrender. The memorial has been designed by Mr. Frederick Wheeler, F.R.I.B.A., and executed by the Coalbrookdale Company.

Dr. Frederick Taylor, senior physician of the hospital and chairman of the memorial committee, called upon General Sir Richard Harrison, who is a governor of the hospital, to unveil the memorial.

General Sir Richard Harrison then unveiled the fountain, and said that memorials of this kind were put up for two reasons. One reason was as consolation to the relatives of those who died, by recognition of the gallant work done by these brave and devoted men in the service of their country. The second reason was to serve as a reminder to all those who passed by that the path of duty lay straight before them. General Harrison handed over the fountain in trust to Mr. Cosmo Bonsor, the treasurer of the hospital.

Mr. Bonsor, on behalf of the President of the hospital (the Prince of Wales) and the Governors, said he accepted the memorial to hold it in trust for ever, and further undertook that a full report of the day's proceedings should be inserted in the minutes of the next General Court of Governors, so that no doubt might ever arise as to the object for which the memorial was presented, or as to the nature and intention of the trust which he thus accepted.

A short service was subsequently held in the hospital chapel.

The next function was the opening of the "Wills Library"

by Sir Frederick Wills, M.P., who had given the governors a sum of at least £6,000 for this purpose. The new library occupies the whole of the ground floor of a building adjacent to the new physiological department, the two forming part of a scheme for remodelling the school buildings and museums. All the bookcases and panellings are of carved oak, and are arranged to form bays for students' work, each bay being lighted by a separate window and containing a large oak reading-table. There is accommodation for some 10,000 volumes. The library is lighted by electricity and heated by water radiators. The work has been carried out by Messrs. Holloway Brothers, under the supervision of Mr. J. H. T. Woodd, the hospital architect, who drew up the plans.

After Sir Frederick Wills had formally opened the library the governors and staff of the medical school of the hospital proceeded to the physiological theatre, where the distribution of medals and prizes to students took place in the presence of a large company of spectators.

The whole assembly subsequently adjourned to the grounds, where a large garden party was held, at which the band of the 1st Life Guards played.

